# A Note on Suckling Behavior and Laterality in Nursing Humpback Whale Calves from Underwater Observations

**DOI:** 10.3390/ani7070051

**Published:** 2017-07-18

**Authors:** Ann M. Zoidis, Kate S. Lomac-MacNair

**Affiliations:** Cetos Research Organization, 11 Des Isle Avenue, Bar Harbor, ME 04609, USA; klomacmacnair@gmail.com

**Keywords:** humpback whales, Megaptera novaeangliae, calf, nursing, behavior, suckling, laterality

## Abstract

**Simple Summary:**

Nursing in large baleen whales and specifically the humpback whale is not well documented and difficult to capture in real time. Using underwater video documentation techniques, we collected digital video footage of humpback whale nursing events. From this, we provide an enhanced descriptive account of humpback whale suckling and recorded milk in the water near a nursing mother and calf column for the first time. As part of our investigation into nursing behaviors, we assessed if humpback calves demonstrated any patterns of laterality during nursing events. A pattern of laterality was noted in that all suckling events had a right side bias. Nursing bouts were short and intermittent, which coincides with what is known of larger terrestrial mammals such as the African elephant and other baleen whales. These shorter nursing periods are likely are due to the energetics of baleen whale milk coupled with the calf′s short respiration cycle before it has to return to the surface to breathe. Our study shows that underwater observations in marine mammal science provide valuable insight into real time events not easily accessible from vessel or aerial platforms.

**Abstract:**

We investigated nursing behavior on the Hawaiian breeding grounds for first year humpback whale (*Megaptera novaeangliae*) calves. We observed and video-documented underwater events with nursing behavior from five different whale groups. The observed nursing events include behaviors where a calf positions itself at a 30–45° angle to the midline of the mother’s body, with its mouth touching her mammary slit (i.e., suckling position). On two occasions, milk in the water column was recorded in close proximity to a mother/calf pair, and on one occasion, milk was recorded 2.5 min after suckling observed. Nursing events, where the calf was located in the suckling position, were found to be short in duration with a mean of 30.6 s (range 15.0–55.0, standard deviation (SD) = 16.9). All observations of the calf in the suckling position (*n* = 5, 100%) were with the calf located on the right side of the mother, suggesting a potential for right side laterality preference in the context of nursing behavior. Our study provides insight into mother/calf behaviors from a unique underwater vantage. Results supplement previous accounts of humpback whale nursing in Hawaiian waters, validate mother/calf positioning, document milk in the water column, and introduce the potential for laterality in nursing behavior for humpback whale calves.

## 1. Introduction

Nursing behavior by humpback whales has been previously described from various surface or in-water platforms. Literature containing descriptions of humpback whale nursing behavior includes in Williamson (1961) [1], Bauer (1986) [2], Clapham & Mayo (1987) [3], and Morete et al. (2003) [4] either directly or indirectly via surface whale observations, and in Glockner & Venus (1983) [5] Glockner-Ferrari & Ferrari (1985) [6] and Cartwright (2005) [7] directly from in-situ underwater studies. Williamson (1961) first reports an observation of surface nursing behavior from a winter sighting of a humpback mother/calf pair on the Grand Bank of Newfoundland; the mother was on her side at the surface [1]. Clapham & Mayo (1987) also described suckling in humpback whales on the feeding grounds [3]. Both these descriptions are likely from older calves since they are from their feeding grounds vs. observations from the breeding grounds within the first few months of birth [2,4]. Clapham and Mayo (1982) used associated surfacing behaviors versus from direct observations [3]. They describe nursing behavior as occurring when the mother was swimming slowly or was stationary at the surface and when the calf rose to breathe on alternate sides of the mother’s caudal peduncle. Bauer (1986) described nursing taking place when the mother was in a rostrum up vertical position and occasionally [2], as with Morete et al., 2003, at the surface with the mother′s flukes extending into the air [4]. Glockner & Venus (1983) delivered the first description of Hawaiian humpback whale nursing behaviors from underwater observations, describing first year calves suckling while the mother was stationary, resting in a horizontal position, at a depth of 10–15 m [5]. This study provided an account of calf nursing position. It notes the calf was positioned vertically, with its head up, positioned below the tail of the mother, and with its mouth pointed at the mammary groves and its flippers pointed forward [5]. A later paper further described calf position at approximately 30° angle to the midline of the mother’s body [6]. Cartwright (2005) provided a similar description with the mother motionless at depth in the water column (10–20 m) with the calf under the mother′s rostrum (under the base of the pectoral fin or under the caudal peduncle or fluke of the mother) [7].

Animal laterality, first introduced by Rogers and Anson (1979) when conducting research on lateralization of the chicken forebrain, has since been studied in a diverse set of species [8]. Laterality is believed to reflect functional asymmetry of the brain, with a corresponding relationship of side preference to either social behaviors or foraging. Laterality has been assessed in only a few marine mammal species, and only one of those directly addresses nursing [9]. While portions of this study were later refuted (e.g., the possibility of nasal suckling; [10]), the basic nursing temporal and spatial behaviors of sperm whale calves were presented.

A recent study comparing various marine and terrestrial mother/young mammal and marsupial pairs [11] found consistent patterns of lateralization in all species examined. They compared data from species including the horse (*Equus ferus caballus*), Pacific walrus (*Odobenus rosmarus divergens*), Siberian tundra reindeer (*Rangifer tarandus sibiricus*), saiga antelope (*Saiga tatarica tatarica*), muskox (*Ovibos moschatus*), eastern grey kangaroo (*Macropus giganteus*), red kangaroo (*M. rufus*), beluga whale (*Delphinapterus leucas*), and killer whale (*Orcinus orca*), and included data from single observations (*n* = 1) for wild argali (*Ovis ammon*) and southern right whales (*Eubalaena australis*) (right whale data were obtained from photographs) [11]. This recent study found consistent right hemisphere dominance for social behaviors and processing, concluding that the evidence was clear that lateralized positioning is beneficial in mother–infant interactions in that it contributes to fitness in the young of the species [11]. Asymmetries in many species are well summarized in Rogers et al., (2013) in which vertebrate lateralization is thoroughly examined and analyzed in great detail over a multitude of terrestrial species [12].

Findings in Karenina et al., 2017 demonstrates that lateralization regarding spatial position near the mother in young occurs in a diverse range of vertebrate species [11]. Other mother/calf cetacean lateralization studies have been presented for beluga whales [13,14] and killer whales [15,16] with similar findings; these studies showed that social behaviors correspond to the right brain hemisphere and foraging behavior corresponds to the left brain hemisphere. Findings from a laterality study conducted on adult wild Indo-Pacific bottlenose dolphin (*Tursiops aduncus*) found that during inquisitive approaches to a human observer (diver), the dolphin used the left eye significantly more frequently than the right eye, and rubbing was conducted significantly more frequently with the left flipper than with the right flipper [17].

The first published laterality study [8] assessed foraging behaviors in chickens and concluded that foraging was performed either entirely or to a greater extent by the left hemisphere (agreeing with the findings on left brain correlations from the Karenina studies). The role of the right hemisphere in social behavior was first shown in young chickens [18]; the right hemisphere of chicks also showed an advantage in tasks with a spatial component. Rogers et al. (2013) [12] and MacNeilage et al. (2009) [19] both examine the evolutionary origins of specialization in the two hemispheres of vertebrate brains. MacNeilage et al. (2009) posited that the left hemisphere originally was involved with patterns of behavior whereas the right was for detecting and responding to unexpected stimuli [19]. MacNeilage (2013) [20] went on to investigate asymmetries in marine mammals and to compare that to trends of laterality in humans. They found biases not unlike human handedness and that marine mammals, humans, and other primates all may have a left hemisphere specialization for spatial action and movement [20]. MacNeilage (2014) [21] further examined a “vertebrate-wide tendency toward a rightward action asymmetry associated with routine behavior,” which they found in both marine mammals, primates, and humans, though the functional origins of the asymmetries may differ [21].

Studying behavioral lateralization of mother/calf whales could provide a mechanism to better understand cetacean brain function, social interactions, and development of young. There are accounts of foraging laterality behaviors in humpback whales on the feeding grounds in which behavioral asymmetries in feeding adults [22,23] and “younger” whales [23] were documented. In the only marine mammal laterality study of nursing behaviors, Gero & Whitehead (2007), found a left-side bias and strong lateral asymmetry in suckling attempts in sperm whale calves (*Physeter macrocephalus*) [9]. They suggested that this may correspond with the theory that the right eye and left-brain correspond with foraging behaviors.

The primary objectives of our study were to (1) provide a video-documented and enhanced descriptive account of humpback whale suckling events from underwater observations, (2) corroborate and expand upon previous accounts of humpback whale nursing behavior, (3) record nursing events in association with milk in the water column for a first time, and (4) investigate patterns if any of laterality among mother/calf pairs of humpback whales during nursing events.

## 2. Methods

We conducted underwater observation and acoustic recording studies of humpback whales on wintering grounds mainly in waters off southwest Maui, Hawaii, in most years from 2001–2015 from a small (7 m) rigid twin hull catamaran motorboat. Data were gathered by 1–3 snorkeling free-divers. Free-divers used video camera assemblies in custom underwater housing consisting of digital Panasonic video cameras (PV-GS200 with a digital high definition 0.45× wide angle lens with macro, and a PV-GS300 with a digital high definition 0.5× wide angle lens with macro). Hydrophones systems were a combination of a single omnidirectional hydrophone (Cetacean Research Technology SQ26-06 hydrophone custom mounted and cabled to 1 m below the camera) and a custom designed (Adam Frankel, Cornell Lab of Bioacoustics) two-element hydrophone array with two HTI MIN-96 hydrophones mounted 1.5 m apart on a bar perpendicular to the optical axis of the camera. Standardized methods for in-water sampling were followed from previously published humpback whale studies [5,24,25,26,27,28].

Surface observation notes were taken of all surface and underwater focal bouts and photographs were taken with a Canon 10D digital camera and 300 mm lens by 1–2 vessel-based observers. Focal sessions focused primarily on any groups with a mother and calf to assess under- and above-water behaviors, group associations, laterality, and underwater vocalizations using sampling methods described in detail in Zoidis et al., 2008 [26] and Zoidis et al., 2014 [27]. Prior to initiating a focal session, baseline observations were made to examine if behavioral states differed before and after free-divers entered the water. Nursing and milk observations along with other behaviors of interest were documented in real time when free-divers returned to the vessel after each underwater bout. Underwater video of all focal sessions was reviewed *post-hoc*, and behaviors were transcribed, including individual group behavior states, movement of the calf relative to the resting mother, surfacing intervals, and calf activities such as play, rest, approach, etc. Duration (in seconds), frequency, and position of the calf relative to the mother were recorded for each observed nursing event from video, photographs, and written and audio notes collected in the field. Same year mother calf pairs were uniquely identified by consistent markings on either animal, particularly from fluke, dorsal fin, or body markings of the mother confirmed by photographs and video documentation.

Group composition is defined as mother/calf or mother/calf/escort (or more than one escort) where an escort is an accompanying adult whale in a discreet spatial association to the mother/calf pair and also showing synchrony of movement [5]. Calves were identified based on size, typically ranging from 1/4 to 1/2 of the length of the proximate adult (presumed mother) in closest association [5,29]. Where possible, mothers were identified by sexing the adult animal closest to the calf from direct underwater observations; if this was not possible, the identity of the mother was inferred from social role or because she was the adult continually observed in close proximity to the calf [5,30,31,32].

We used the definitions of nursing behavior in wintering humpback first year calves following that of Glockner & Venus (1983) [5] and Glockner-Ferrari & Ferrari (1985) [6] where nursing occurred at depth (10–15 m) during which a calf approached the resting (stationary) mother’s peduncle area, dove beneath her, positioned its rostrum under the mother’s belly close to her mammary slit located on her ventral side behind her dorsal fin at the peduncle, at an angle of approximately 30–45° from the midline of the mother. A nursing event was defined as a period >5 s in which the calf remained in the sucking position described above (i.e., presumed that the calf is locked on to the mother’s nipple) [33]. During these nursing events, the mother could be vertical or horizontal and the calf’s suckling position would vary based on the mother’s resting angle. We define laterality in this study as an asymmetry with regard to a preference shown for behaviors occurring on one side of the body vs. the other (i.e., the calf nursing on one side of the mother more than the other).

## 3. Results

We reviewed underwater digital video recordings from 10 of our winter study seasons of 199 focal groups in which a calf was present (1485.5 min; mean = 7.5 ± 9.0 min per focal group; range = 1.0 to 53.5 min). From this video dataset we found five occasions (3%) where nursing behavior and/or milk was observed and video-documented. In these five focal groups, there were four separate nursing events, one with an accompanying observation of milk, and a single event where milk was recorded with no nursing observed (i.e., nursing had likely occurred prior to free-divers entering the water to start focal session). All five focal groups were unique animals.

Focal session duration, duration of calf in suckling position, milk observations, and calf positioning (i.e., calf right or left location relative to the mother during suckling) are provided in Table 1, and a further descriptive analyses of each focal session is provided below. Focal sessions of the five nursing events ranged from 13–48 min in duration. During Focal Group 1 (FG1), two events with the calf in the suckling position were recorded with a period of 5 min between; during Focal Group 3, 4 and 5 (FG3, FG4, and FG5), the calf was observed in the suckling position once. Overall, duration of suckling ranged from 15.0 to 55.0 s, with a mean suckling duration of 30.6 s (SD = 16.9). There were two events where milk was recorded in the water column in close proximity (<5 m) to a focal group and when no other humpback whale groups were observed in the area. During FG2, milk was observed and photographed in the water without the observation of suckling; thus, nursing was presumed to have occurred prior to free-divers entering the water with the mother/calf pair. During FG4, milk was observed and photographed 2.5 min after suckling was observed.

During all groups where the calf was observed in the suckling position, the calf was located on the right side of the mother, and none were observed with the calf located on the left side. The calf approached the mother on the right on four of the five occasions prior to positioning to suckle. On one occasion, the calf approached the mother from the left and repositioning itself on the right prior to beginning suckling.

**Figure 1:**
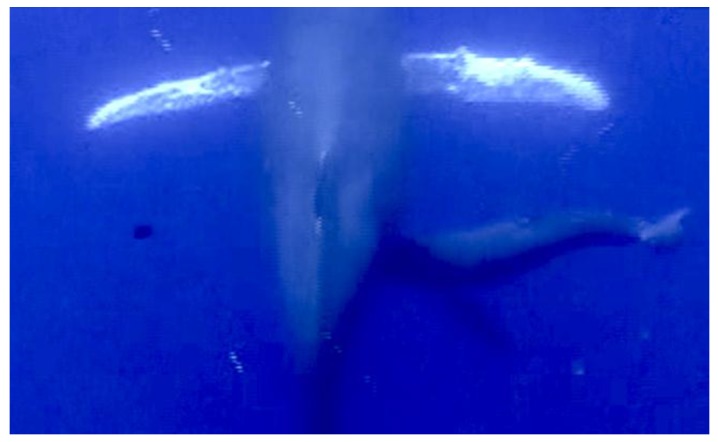
Focal Group 1 where calf approached and positioned on right side, remaining in the suckling position for duration of 55 s.

Focal Group 1: On 25 February 2006 we recorded a mother, calf, and escort group approximately 1 km west of Olowalu, Maui, for 30 min. Two separate sucking events were observed and video-documented during this focal session. FG1 was the only focal group where suckling was observed twice. During the first suckling event, the calf approached the mother on the right side and positioned itself at approximately a 45° angle (Figure 1) while the mother was resting stationary, almost horizontally, approximately 15 m below the surface. After a duration of 55 s with the calf in suckling position, the escort approached from deeper water, on the right side (i.e., with the calf positioned between the escort and mother). At the arrival of the escort, the calf repositioned itself by moving under the belly of the mother, terminating suckling. A second suckling event occurred 5 min later when the escort had again moved to deeper water and was no longer visible. During this second sucking event, the calf again approached the mother from the right side and remained on the mother’s right side in suckling position at approximately a 45° angle for 27 s duration.

**Figure 2:**
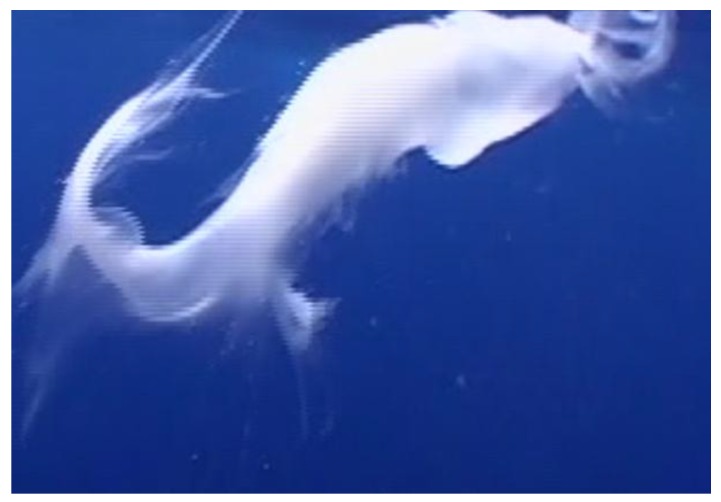
Free floating milk during observation of Focal Group 2.

Focal Group 2: On 1 March 2006, we initially observed a mother/calf pair from our vessel, traveling slowly traveling within 1 km of the island of Lanai. On initiating a focal session, the free-divers, upon entering the water in proximity to the mother/calf pair, observed and video documented streams of milk clearly visible in the water column in and around the two whales as they traveled slowly about 3 m below the water surface. The calf appeared quite young, likely less than two weeks old, estimated at about one-third the mother′s Body Length (BL) and lighter grey in color than older calves. This calf was never more than a distance of one calf BL from the mother and moved between right and left side of the mother throughout the four-min focal session. Underwater video was taken of the milk and whales though no suckling was observed. The milk hung cohesively together in the water column for approximately 90 s as it floated toward the surface and slowly dissipated (Figure 2).

**Figure 3:**
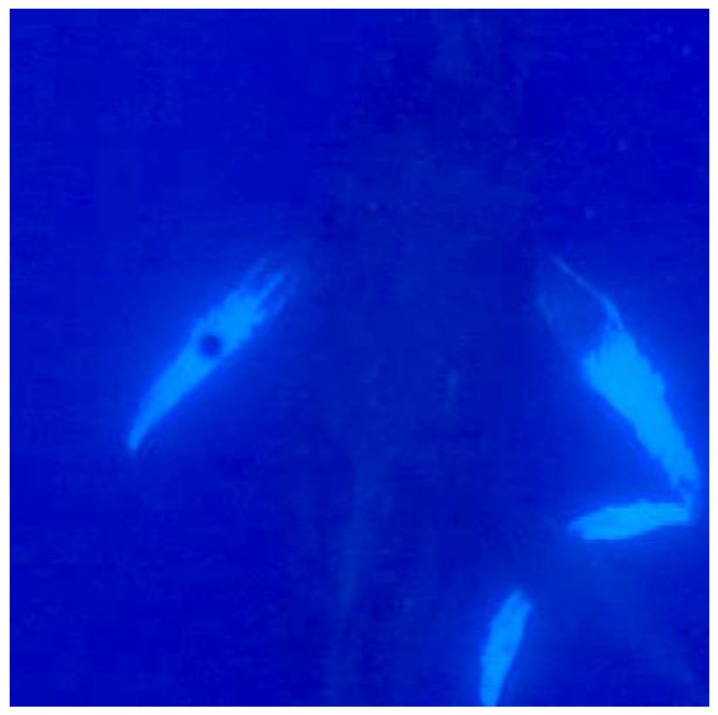
Focal Group 3 where calf approached and again positioned on right side, remaining in the suckling position for duration of 15 s. Both mother and calf sculling with pectoral fins to maintain positioning.

Focal Group 3: On 1 March 2006, we video-documented a mother, calf, and escort group resting in waters approximately 1.5 km northwest of Olowalu, Maui, for 30 min. The mother was observed resting in a nearly vertical position in ca.15 m of water sculling (i.e., moving her pectoral fins to remain in position in the current). After surfacing and diving to return to the resting mother, the calf approached on the right side of the mother. As the calf approached, the mother’s pectoral fins were alongside her body. The calf moved into a sucking position on the right side at approximately a 45° angle. Both the mother and calf began sculling with pectoral fins. The suckling event was 15 s in duration (Figure 3).

**Figure 4:**
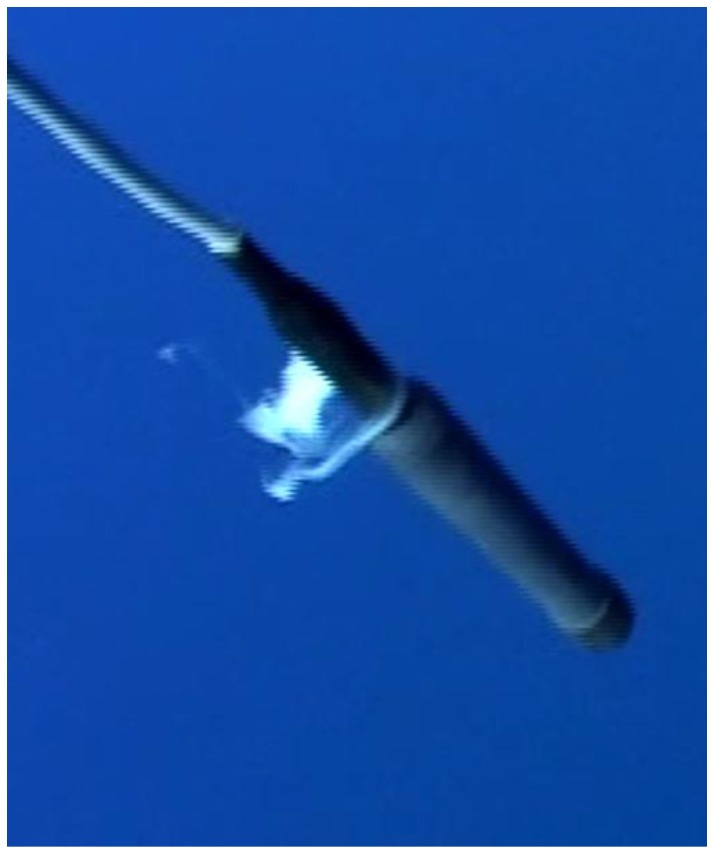
Milk recorded in the water column wrapped around hydrophone (1 m in length).

Focal Group 4: On 15 January 2008, we video-documented a mother and calf for 13 min resting approximately 1.6 km west of Olowalu, Maui. The mother and calf were approximately 15 m below the surface. The mother was observed sculling with her pectoral fins to remain in a semi-vertical (approximately 60°) position. After surfacing, the calf approached the mother on the left side, circled under the mother, and moved to her right. As the calf approached, the mother′s pectoral fins were alongside her body. Once the calf was in nursing position, the mother began sculling again. The suckling event was 16 s in duration. We observed and video documented milk in the water column 2.5 min after the nursing event. The milk hung cohesively together in the water column for less than 1 min then dissipated (Figure 4).

Focal Group 5: On 13 February 2009, we video-documented a mother, calf, and escort group for 48 min resting in waters approximately 1 km southwest of Lahaina, Maui. The mother and calf were ca. 12 m below the surface. The water visibility was more murky than usual. However, the reduced visibility did not prevent our underwater observations. After surfacing, the calf approached the mother, who was resting horizontally on the right side, and moved into the suckling position. The calf remained in the suckling position on the right side for 40 s before re-positioning to rest under the rostrum of the mother. During this nursing event, the escort was not visible—he was possibly in deeper water below the mother/calf pair.

## 4. Discussion

Humpback whale nursing events and the potential of behavioral laterality were examined in the waters of the Hawaiian island breeding and birthing grounds. We set out to build upon the previous body of literature on humpback whale mother and calf nursing positioning in Hawaiian humpback whales and further describe suckling positions and durations in more detail and with direct video documentation. All nursing events documented in our study occurred at depth, below the surface, and none out of the 199 groups observed occurred at the surface. This differs from other humpback whale vessel-based study [1,2,3,4] reports in the literature for nursing in humpback whales. None of these papers provide detailed accounts of nursing or photographic documentation, so it is difficult to draw comparisons between the surface observed nursing events in these previous studies and the underwater nursing events in our study or the other Hawaii-based underwater studies that did not cite these behaviors.

Our observations and descriptions of underwater suckling behaviors support and expand upon those reported by Glockner & Venus (1983) [5] Glockner-Ferrari & Ferrari (1985) [6], and Cartwright (2005) [7] in the same humpback population. These previous in-water studies conducted off Maui via underwater observations found that during nursing events, the mother remains stationary or travels slowly, that nursing occurs with calf in a vertical or semi-vertical position, at 10–15 m depth, the calf positioned below the mother with its mouth touching her mammary slit in a suckling position with calf positioning typically preceded by a position under the mother’s caudal peduncle or flukes [5,6,7]. We had similar findings in our study, with additional detail. We video documented mothers oriented angled vertically (nose up and tail down at depth) or nearly horizontally. Nursing orientation of the calf varied with the mother’s resting angle, though generally the calf was oriented semi-vertically with nose up and tail down at depth as previously reported. In one encounter where the mother was sculling, the calf was documented exerted effort to stay in nursing position by sculling its own pectoral fins. We found that escort appearance and presence had potential influence on calf positioning and on suckling duration. During FG1, the escort joined the mother/calf pair approaching from deeper water appeared to be the causative factor in the calf to terminate suckling. This calf re-positioned and did not resume suckling until 5 min had passed and until the escort was no longer visible to the free-diver, indicating it had left or returned to deeper water.

We report the first video documentation of milk in the water column and simultaneous positioning with the calf suckling, which to our knowledge has not been previously described or photographed. The incidences of milk in the water in proximity to a humpback mother/calf pair provided the rare visual evidence (beyond calf positioning) that suckling and the transference of milk was indeed occurring. Previous surface-based or other underwater studies could only presume nursing was occurring from the calf’s position; this milk sighting confirms that nursing is actually occurring.

The occurrence of observed nursing was rare and infrequent (3% of the 199 focal sessions over the 10 years of the study). However, this is a similar percentage of nursing bouts, albeit over a range of species as number of nursing observations, cited in Smultea et al., 2017 [34] (2.5% out of 160 groups). Possible reasons for the small number of nursing observations include large whales may nurse intermittently and briefly, or at depth where observations cannot be made from any platform.

Our study found that periods where the calf was in suckling position were brief (15–55 s) in duration. The intermittent and short nature of nursing behavior we noted corresponds to what has been found in other large whales (Table 2).

Studies on another large mammalian species, such as the African elephant (*Loxodonta africana*), with calves both in captivity and the wild yielded similar results regarding nursing bout brevity. The average duration of a wild African elephant calf′s nursing bout was 86 s [37]. Elephant calves in the wild are estimated to suckle at a rate of only 170 s/h for males and 130 s/h for females [38]. Andrews et al. (2005) found the average duration of suckling for a male calf was 76 s for captive African elephant calves [39].

The evidence from other cetacean and another large mammalian terrestrial species also having very short nursing events points to a likely energetic correlation as yet not understood, with some overarching benefit bestowed by short nursing events. There may be an added constraint in the case of whales due to an association between underwater nursing and the calf’s short respiration interval. If suckling at depth is energetically costly for cetacean calves, it could not be sustained for long periods, since young calves must breath frequently. Calf respiration intervals lengthen, as the calf gets older over the course of the first breeding season. However, they do not generally exceed approximately 3 min in younger calves and up to 4.5 min in older calves [28]. Frequent surfacings result in energy consumption; it takes energy to dive down to the mother and to return to the surface. Such a short breathing interval would be further reduced by any additional energetic consumption at depth from e.g., sculling, such as we observed in 2006. Thus, short nursing bouts are what we would expect given the number of other factors involved. Alternatively, rates of milk transfer in larger mammals, including in whales, are high [40], and the calf’s energetic needs may be met quickly. It is likely that nursing event durations increase in duration as the calf matures and develops breath-holding capacity, as noted in southern right whale calves [35].

All five suckling events showing a right side bias suggested humpback whale calves have laterality preference in the context of nursing behavior. Laterality studies in adult baleen whales during summer (foraging) periods found humpback whales showed some degree of behavioral asymmetries and a general right side bias [22,23]. Foraging grey whales were also shown to have a right side foraging bias [41]. Other laterality studies have focused on side biases shown during travel vs. nursing. In the most recent and comprehensive species comparison of laterality [11], they found consistent right hemisphere dominance for social behaviors and processing, including in mother/young marine mammals. Our study agrees with these findings. A previous study on laterality of beluga whale mother/calf pairs [13] found significant behavioral laterality where beluga calves swam or rested on the right side of the mother and for significantly longer periods than on the left side. Karenina et al. (2013b) [15] found laterality bias in killer whale mother infant pairs in travelling whales, with a group-wide preference for the calf on the mother’s right side when animals were further from the observation vessel, congruent with findings from beluga studies [13,14]). Overall, during avoidance situations, the killer whale calf tended to be on the left, and more often on the right during socializing. A study on adult wild Indo-Pacific bottlenose dolphin [17] had similar visual discrimination results to the Rogers and Anson (1979) chicken study [8]; during inquisitive approaches to an underwater diver, the dolphin used the left eye significantly more frequently than the right eye. However, during social behaviors, the flipper to body contact/rubbing was found to be significantly more frequent and longer with the left flipper than right flipper. The focus of “handedness” as it might relate to marine mammal appendages (flippers) is intriguing and will be addressed in our future studies.

These apparent preferences are considered likely to be indicative of a functional significance to laterality and that the bias varies depending on context. Further study is needed to discern the determining factors for the right side nursing and potential for nursing bias noted in this study. Additional investigation should include better understanding of (1) whether or not the lateral positioning is dictated by the mother or initiated by the calf, (2) if the bias is socially or physiologically (brain hemisphere and/or visual field) driven, and (3) whether the asymmetry seen in Hawaiian humpback whales is found at a population level.

## 5. Conclusions

Our study highlights the importance of direct observational documentation in a natural setting for cetaceans as a method to corroborate nursing and other behaviors and as a way to quantify behaviors that previously were descriptive or anecdotal accounts. Direct observer free-diver studies in cetaceans provide valuable insight into identification of social patterns, intraspecific behaviors, and underwater interactions in an important period of humpback whale mother/calf breeding, and provides a basis for future investigations.

## Figures and Tables

**Table 1:** Summary of nursing events and milk observations.

Date	Focal Group	Group Composition	Focal Session Duration (min)	Suckling Observed	Milk Observed	Suckling Duration (s)	Calf Position During Suckling	Calf Approach Side	Figure Number
2/25/06	FG1 *	M/C/E	30	Yes	No	55, 27	R, R	R	1
3/1/06	*FG2*	M/C/E	4	No	Yes	-	NA	NA	2
3/1/06	*FG3*	M/C/E	30	Yes	No	15	R	R	3
1/15/08	*FG4*	M/C	13	Yes	Yes	16	R	L	4
2/13/09	*FG5*	M/C/E	48	Yes	No	40	R	R	NA

* Suckling was observed twice during focal session; M/C/E = Mother, calf, and escort, M/C = Mother/calf pair, R = Right, L = Left.

**Table 2:** Summary of suckling duration previously recorded during other large whale studies.

Species	Suckling Duration (s)	Reference
Southern right whales	90–210	Thomas & Taber 1984 [35]
Bowhead whale	<60	Wursig et al., 1984 [36]
Gray whale	20–76	Smultea et al., 2017 [34]
Blue whale	13–264	Smultea et al., 2017 [34]
Fin whale	30–138	Smultea et al., 2017 [34]
